# Synthesis of quenchable amorphous diamond

**DOI:** 10.1038/s41467-017-00395-w

**Published:** 2017-08-22

**Authors:** Zhidan Zeng, Liuxiang Yang, Qiaoshi Zeng, Hongbo Lou, Hongwei Sheng, Jianguo Wen, Dean J. Miller, Yue Meng, Wenge Yang, Wendy L. Mao, Ho-kwang Mao

**Affiliations:** 1 Center for High Pressure Science and Technology Advanced Research (HPSTAR), Shanghai, 201203 China; 2 HPSynC, Geophysical Laboratory, Carnegie Institution of Washington, Argonne, IL 60439 USA; 3 Center for Nanoscale Materials, Argonne National Laboratory, Argonne, IL 60439 USA; 4 HPCAT, Geophysical Laboratory, Carnegie Institution of Washington, Argonne, IL 60439 USA; 5 Department of Geological Sciences, Stanford University, Stanford, CA 94305 USA; 6 Stanford Institute for Materials and Energy Sciences, SLAC National Accelerator Laboratory, Menlo Park, CA 94025 USA; 7 Geophysical Laboratory, Carnegie Institution of Washington, Washington, DC 20015 USA

## Abstract

Diamond owes its unique mechanical, thermal, optical, electrical, chemical, and biocompatible materials properties to its complete *sp* ^3^-carbon network bonding. Crystallinity is another major controlling factor for materials properties. Although other Group-14 elements silicon and germanium have complementary crystalline and amorphous forms consisting of purely *sp* ^3^ bonds, purely *sp* ^3^-bonded tetrahedral amorphous carbon has not yet been obtained. In this letter, we combine high pressure and in situ laser heating techniques to convert glassy carbon into “quenchable amorphous diamond”, and recover it to ambient conditions. Our X-ray diffraction, high-resolution transmission electron microscopy and electron energy-loss spectroscopy experiments on the recovered sample and computer simulations confirm its tetrahedral amorphous structure and complete *sp* ^3^ bonding. This transparent quenchable amorphous diamond has, to our knowledge, the highest density among amorphous carbon materials, and shows incompressibility comparable to crystalline diamond.

## Abstract (web summary)

Diamond’s properties are dictated by its crystalline, fully tetrahedrally bonded structure. Here authors synthesize a bulk *sp* ^3^-bonded amorphous form of carbon under high pressure and temperature, show that it has bulk modulus comparable to crystalline diamond and that it can be recovered under ambient conditions.

## Introduction

Bonding and structure determine the properties of materials. Carbon has numerous allotropes with diverse bonding (*sp* ^1^-, *sp* ^2^- and *sp* ^3^-hybridized bonds), structures, and corresponding properties. Among them, diamond is a unique crystalline phase with fully *sp* ^3^-hybridized C-C bonds. The extremely strong directional *sp* ^3^ bonds lead to many extraordinary properties and applications of diamond, such as the highest known hardness, very low friction and adhesion, unmatched thermal conductivity, electronic mobility, the highest electron dispersion, high dielectric breakdown, radiation hardness, biocompatibility, and chemical inertness^1, 2^. Moving away from the long-length-scale perfect ordering of single-crystal diamond, nanocrystalline diamond (grain size 10–200 nm) shows higher hardness and stiffness than monocrystalline or polycrystalline diamond^3, 4^. Nanotwinned diamond (twin thickness ~5 nm) exhibits even higher hardness and thermal stability than nanocrystalline diamond^5^. Ultra-nanocrystalline structure (grain size 2–5 nm) facilitates high n-type conductivity in diamond, which is otherwise unattainable^6^. Naturally it is very interesting to reach the extreme end of order where translational periodicity completely disappears (i.e. purely *sp* ^3^-bonded tetrahedral amorphous carbon). This carbon form is expected to have unique properties combining the advantages of crystalline diamond and amorphous materials and even beyond.

Although purely *sp* ^3^-bonded tetrahedral amorphous carbon is of great interest, it has not yet been observed. Considerable efforts have been devoted to developing amorphous carbon with high *sp* ^3^ fractions (up to 88%), named diamond-like carbon^7–9^. These materials show terrific mechanical properties and have been widely used as protective coatings^8^. However, they are only available as thin films (with typical thicknesses ranging from a few to tens of nanometers), and still contain considerable amounts of *sp* ^2^ bonds. In contrast, other Group-14 elements silicon and germanium both have *sp* ^3^-bonded amorphous and crystalline polymorphs. The *sp* ^3^-bonded tetrahedral amorphous silicon (*a-*Si) and germanium (*a-*Ge) have been known for decades and have widespread applications in photovoltaics, displays, and flat-panel detectors^10, 11^. Therefore, synthesis of bulk amorphous carbon with purely *sp* ^3^–bonded network like diamond has been a long-standing pursuit.

Pressure is a powerful tool to induce *sp* ^2^-*sp* ^3^ bonding transition in carbon allotropes including graphite, carbon nanotubes, fullerenes, and glassy carbon, etc^12–15^. Among them, the transition in glassy carbon is particularly interesting. Glassy carbon is an amorphous carbon allotrope consisting primarily of *sp* ^2^-bonded carbon^16^. Compression of glassy carbon to 44 GPa or higher pressure at room temperature was reported to result in a high-pressure amorphous carbon phase with *sp* ^3^ bonding and superior strength^14, 15, 17^. However, in contrast to the tetrahedral network structure in *a-*Si and *a-*Ge, this high-pressure phase still maintained a layered structure quite similar to the starting glassy carbon material according to its X-ray diffraction (XRD) patterns, and the pressure-induced *sp* ^2^-*sp* ^3^ transition was reversible upon decompression. On the other hand, irreversible transitions in carbon materials can usually be realized by combining high pressure with high temperature (HPHT). For instance, by using large volume presses (LVP) or shockwaves, crystalline diamonds (bulk or nano) have been synthesized from various *sp* ^2^-bonded carbon allotropes through an irreversible *sp* ^2^-*sp* ^3^ transition under HPHT (typically 15–25 GPa, 2100–2800 K)^3, 5, 18, 19^. In addition to crystalline diamond, other carbon forms, such as a disordered carbon phase with both *sp* ^2^ and *sp* ^3^ bonds were synthesized in LVP^20^. The resulting product is highly dependent on the starting material and the pressure-temperature (P-T) ranges used in the experiments. Therefore, exploring carbon materials over a broader P-T range is a promising approach to produce novel quenchable carbon forms.

In this study, by using a diamond anvil cell (DAC) coupled with in situ laser heating, we explore a P-T range rarely studied before for the carbon system. Using glassy carbon as a starting material, we synthesize an *sp* ^3^-bonded tetrahedral amorphous carbon which can be recovered to ambient conditions, i.e. quenchable amorphous diamond. The structure, bonding, and properties of quenchable amorphous diamond are investigated using XRD, high-resolution transmission electron microscopy (HRTEM), electron energy loss spectroscopy (EELS), and ab initio molecular dynamics (MD) simulation. Amorphous diamond is optically transparent, dense, and shows ultrahigh incompressibility (bulk modulus) comparable to crystalline diamond.

## Results

### Sample synthesis and synchrotron XRD

**Fig. 1:**
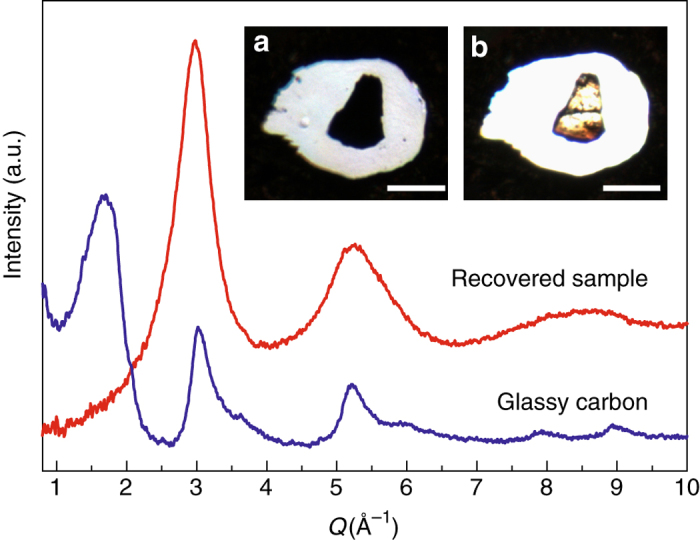
Structure and image of the recovered sample. XRD patterns of the starting material glassy carbon (*blue*) and the sample recovered from high pressure and temperature (*red*). *Inset* **a**, Optical microscope image of the sample in the DAC at 49.6 GPa before laser heating showing the sample is opaque. *Inset* **b**, The sample becomes optically transparent after laser heating at the same pressure. The *scale bars* in insets represent 50 μm. The sample size shrinks by approximately 3% in length and width, leading to an estimated volume decrease of nearly 10%, assuming the sample thickness also decreases by 3%

Glassy carbon samples were compressed to 50 GPa at room temperature in a DAC, followed by laser heating at approximately 1800 K until the sample became transparent. This optimized temperature avoids crystallization at higher temperatures and incomplete conversion at lower temperatures. After quenching to room temperature and releasing pressure, we removed the recovered samples from the DACs for further investigation (see “Methods” section for details). The starting glassy carbon sample consists of curved carbon sheet fragments^16^. In its XRD pattern, the first sharp diffraction peak (FSDP) at ~1.7 Å^−1^ and the second diffraction peak at ~3.0 Å^−1^ originate from the interlayer and intralayer distances of the curved carbon sheets, respectively (see Fig. 1). In contrast, for the recovered sample, the characteristic intense FSDP in glassy carbon at 1.7 Å^−1^ is absent, indicating that the layered structure has disappeared. Moreover, the peaks at ~3.0 Å^−1^ and in a higher *Q* range are replaced by broad, highly diffuse peaks. These dramatic changes suggest the recovered sample has a totally different structure from the initial glassy carbon. XRD patterns also clearly distinguish the recovered amorphous sample from nanocrystalline diamond with an average grain size as fine as 2 nm, as estimated by the Scherrer equation (Supplementary Fig. 1). The first peak of the recovered sample has a full width at half maximum of 0.63 Å^−1^, close to that of *a*-Si (~0.6 Å^−1^) with a random *sp* ^3^-bonded network^10^.

### Density of the recovered sample

The altered structure of the recovered sample results in a dramatic change in its properties compared with glassy carbon. The starting glassy carbon sample remains opaque when compressed to ~50 GPa (Fig. 1a), consistent with previous results^17^. However, after laser heating at the same pressure the sample becomes optically transparent (Fig. 1b). At the same time, the sample volume shrinks by nearly 10% after heating according to the microscope images (Fig. 1a, b). While the volume of unheated glassy carbon reduces greatly during compression but mostly recovers after decompression, the volume of the new transparent sample expands little during decompression, indicating the recovered sample has a much higher density than glassy carbon. Although measurement of density is difficult for the high-pressure samples which are typically tens of micronmetres, we were able to estimate the density of these samples based on their plasmon peak energy derived from low loss EELS^21, 22^. Given the plasmon energy of 31.8 eV (Supplementary Fig. 2), we estimated the density of the recovered sample to be 3.3 ± 0.1 g cm^−3^ (atomic density 0.166 ± 0.005 atom Å^−3^), which is close to the density of diamond (3.52 g cm^−3^).

### Atomic structure and chemical bonding

**Fig. 2:**
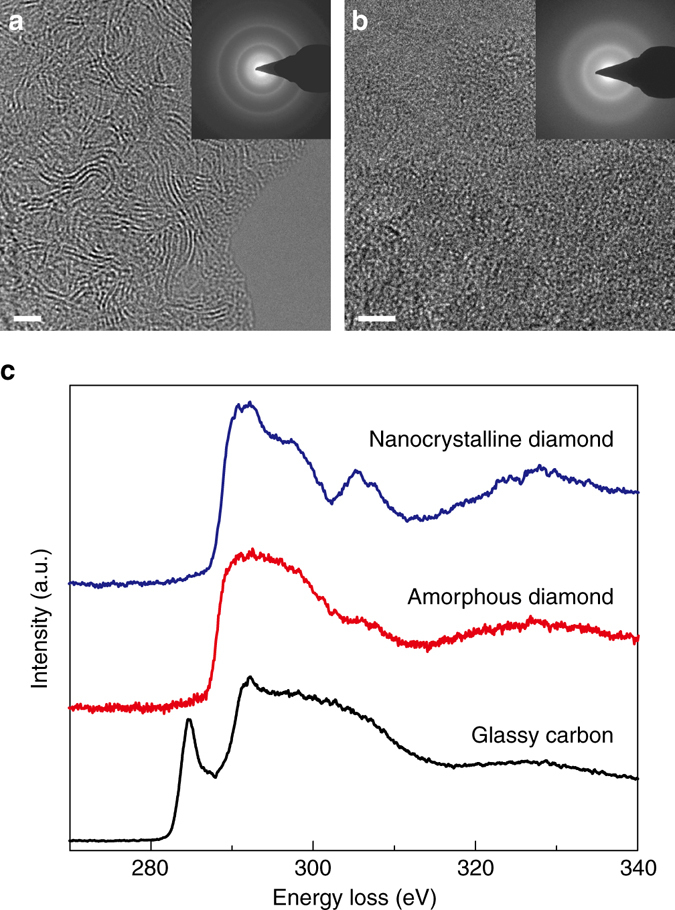
TEM studies of the recovered sample. **a** HRTEM and selected area electron diffraction (*SAED*) images of glassy carbon **b** HRTEM and SAED images of the recovered amorphous diamond sample revealing the amorphous structure. The *scale bars* in **a** and **b** represent 2 nm. **c** Carbon K-edge EELS of glassy carbon (*black*), the recovered amorphous diamond sample (*red*) and nanocrystallline diamond (*blue*). The fine post-edge structures in the EELS of nanocrystalline diamond, e.g. the large dip at ∼302 eV which reflects the second absolute band gap, are characteristics of crystalline diamond. Their absence in the EELS of amorphous diamond is further evidence of the disordered atomic arrangement in amorphous diamond. We find a similar phenomenon when comparing the EELS patterns of crystalline and amorphous Si^24^

We further investigated the atomic structure and chemical bonding of the recovered sample in comparison with the starting glassy carbon material using HRTEM (see “Methods” section for details). Consistent with our XRD results, the glassy carbon is composed of numerous curved graphene layers with interlayer distances ranging from 3.5–3.9 Å, while the recovered sample no longer shows a layered structure (Fig. 2a, b). The randomly arranged atoms in the HRTEM image and diffuse diffraction rings in the electron diffraction image both indicate that the recovered sample has no identifiable crystalline symmetry. This disordered structure is entirely different from nanocrystalline diamond with extremely small crystals (1–3 nm) (Supplementary Fig. 3). EELS is one of the most reliable tools for probing the bonding in carbon materials^23^. The carbon K-edge EELS of the recovered sample is compared with that of glassy carbon and nanocrystalline diamond in Fig. 2c ^24^. The EELS of glassy carbon shows a sharp pre-peak at ~285 eV that corresponds to π bonding, as a result of its nearly 100% *sp* ^2^ bonds^23^. This pre-peak is not present in the EELS of the nanocrystalline diamond due to its purely *sp* ^3^ bonds. Similarly, the EELS pattern of the recovered carbon sample has no pre-peak, implying its atoms should be fully *sp* ^3^-bonded like those in crystalline diamond. This amorphous carbon form converted from glassy carbon is fully *sp* ^3^-bonded, optically transparent, dense, and is named quenchable “amorphous diamond”. The quenchable amorphous diamond is fundamentally different from the cold compressed “high-pressure amorphous diamond” which still highly resembles the layered atomic structure of the initial glassy carbon characterized by the FSDP at ~1.7 Å^−1^, and cannot be recovered to ambient conditions probably due to the small energy barrier between two similar structures^14^.

### Compressibility of amorphous diamond

**Fig. 3:**
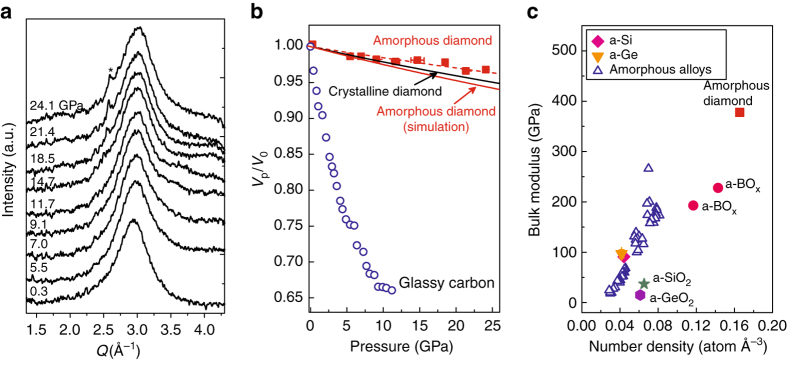
In situ high-pressure XRD of amorphous diamond. **a** XRD patterns of the amorphous diamond sample as a function of pressure. The X-ray wavelength is 0.4066 Å. Diffraction peaks from the pressure medium helium are marked by symbol *. **b** Pressure vs. fractional volume changes for glassy carbon (*circles*)^25^, crystalline diamond (*black solid line*) and amorphous diamond (*squares* for experimental results, *red solid line* for simulation results). The *dashed line* is a second-order BM-EOS fit to the experimental data. The volume change of amorphous materials is estimated by assuming a cubic power law between the volume and the inverse position of the diffraction peak (q^−1^). The peak position was derived by fitting the diffraction peaks to a Gaussian function. **c** Bulk moduli and atomic number densities of amorphous diamond, *a-*Si^10^, *a*-Ge, SiO_2_ glass, GeO_2_ glass^35^, amorphous BO_x_^32^, and amorphous alloys (metallic glasses)^34^. The *error bars* of pressures were estimated by measuring pressure before and after each XRD measurement

Because amorphous diamond consists of *sp* ^3^-hybridized C-C bonds, it is expected to have remarkable mechanical properties. We performed in situ high pressure XRD experiments to investigate the compressibility of the amorphous diamond (see “Methods” section for details). The FSDP of amorphous diamond only shifts slightly to higher angle with increasing pressure from 0.3 GPa to 24.1 GPa, demonstrating it is highly incompressible (Fig. 3a). The volume change of amorphous diamond was estimated by assuming a cubic power law between the volume and the inverse position of the diffraction peak (q^−1^). In comparison to glassy carbon, which is a rather “soft” material (bulk modulus of ~30 GPa)^25^, amorphous diamond shows ultrahigh incompressibility (Fig. 3b). Fitting the experimental data to a second-order Birch-Murnaghan equation of state (BM-EOS) derived its bulk modulus of 624 ± 95 GPa. This extremely high bulk modulus is consistent with its ultrahigh incompressible behavior. However, we need to be cautious about the absolute value of the bulk modulus obtained herein, considering the relatively large uncertainties in fitting the broad weak diffraction peaks of the minute amorphous sample in a DAC. Moreover, as a result of the poor X-ray scattering ability of carbon, the background scattering signal from the pressure medium—helium, which is typically invisible or negligible in most high-pressure experiments, may considerably affect the accurate determination of the diffraction peak position of the amorphous diamond. These uncertainties are also reflected by the relatively scattered experimental data shown in Fig. 3b.

### Ab initio MD simulation

**Fig. 4:**
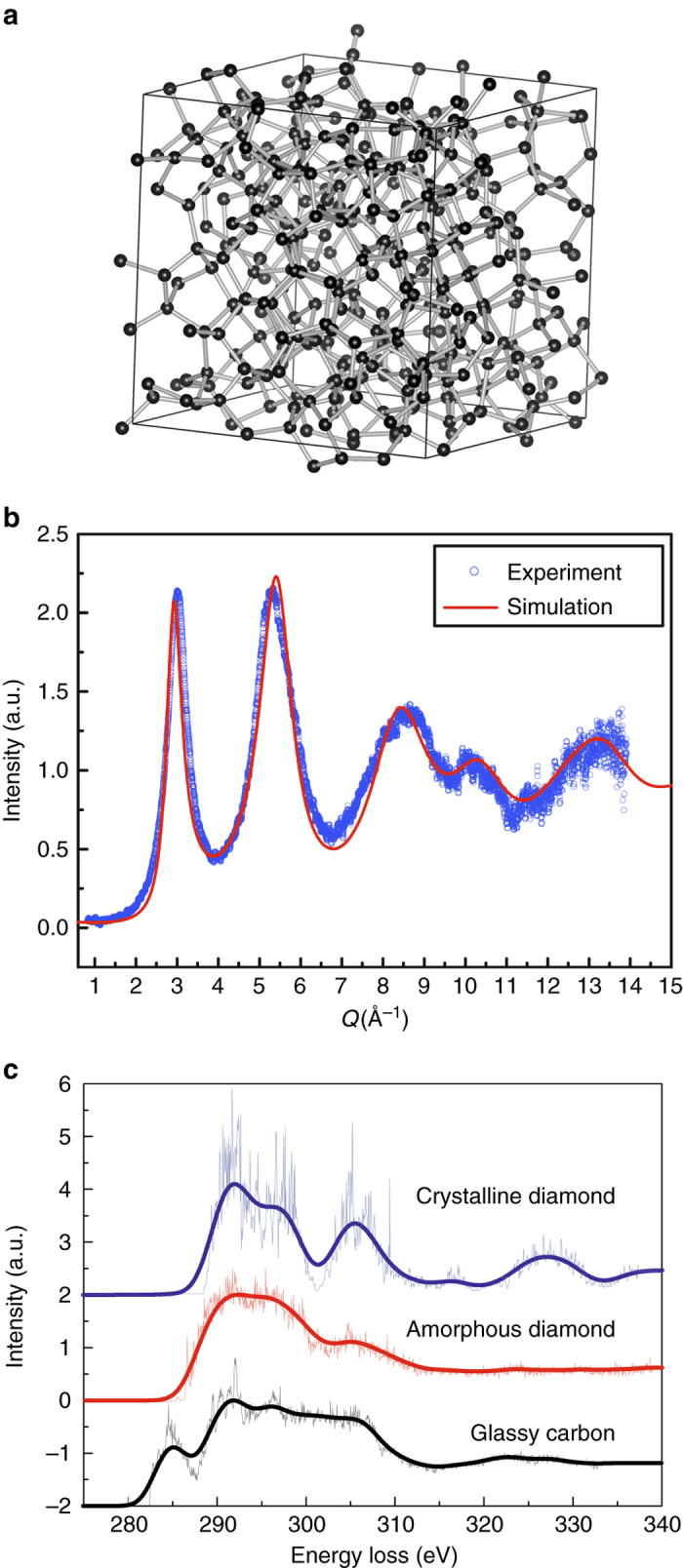
Computational simulation of the amorphous diamond structure. **a** Structural model of amorphous diamond. **b** Calculated XRD pattern (structure factor) of amorphous diamond (*solid red line*) in comparison to experimental data (*open blue circles*). **c** Calculated EELS for crystalline diamond (*blue*), amorphous diamond (*red*), and glassy carbon (*black*). The as-obtained EELS (*thin lines*) were smoothed using a Gaussian smoothing window function with the window width set as 1.2 eV, and the results are shown as *thick lines*

As a result of *sp* ^3^ hybridization, atoms in amorphous diamond are expected to be 4-fold coordinated, forming a randomly oriented network which is highly analogous to the structure of *a-*Si and *a-*Ge^26^. The microscopic structure of amorphous carbon with different *sp* ^3^ fractions has previously been studied by ab initio simulations under conditions close to ambient pressure^27, 28^. In our ab initio MD simulation, we quenched liquid carbon very slowly under high pressure, followed by pressure release to zero pressure (see “Methods” section for details). Figure 4a shows a structural model of the tetrahedral amorphous carbon obtained from simulation, in which carbon atoms are 4-fold coordinated. The coordination number (CN) is calculated to be 3.95 (Supplementary Fig. 4). We also conducted Wannier function analysis to examine the bonding nature in this amorphous carbon structure^29, 30^, and derived the percentage of tetrahedral bonding to be 98% (Supplementary Fig. 5). In terms of bond-angle distributions, the amorphous diamond has a bond-angle distribution function centered at 109.5°. These results suggest a nearly perfect tetrahedral amorphous carbon network. Similarly, a low fraction of dangling bonds was also observed in *a-*Si^31^. The calculated density of this structure (3.28 g cm^−3^) is consistent with experimental data. The XRD pattern (structure factor S(Q)) computed using this model matches the experimental data in peak positions, peak widths and intensities quite well (see Fig. 4b), implying the structure of amorphous diamond synthesized in this work is well described by a random tetrahedral network model obtained by melt-quenching in simulations. In addition, the calculated EELS based on this structural model shows the same features as the experimental EELS of amorphous diamond (Fig. 4c). The π bonding peak at 285 eV is present in glassy carbon, but absent in crystalline or amorphous diamond. Furthermore, in contrast to the characteristic fine post-edge structures in the EELS of crystalline diamond, e.g. the large dip at ~302 eV, the EELS of amorphous diamond only contains broad bands. This is consistent with the disordered atomic arrangement in amorphous diamond. We find a similar phenomenon when comparing the EELS patterns of crystalline and amorphous Si^24^. These results provide further evidence that amorphous diamond synthesized in this study is tetrahedrally *sp* ^3^ bonded. The bulk modulus of the simulated amorphous structure is also derived (*B*_0_ = 377.6(3) GPa, *B*_0_’ = 3.62) by calculating its equation of state (see Fig. 3b and Supplementary Fig. 6), which is only slightly lower than that of crystalline diamond (calculated *B*_0_ = 438.1(7) GPa, *B*_0_’ = 3.60). The bulk modulus and atomic density of amorphous diamond are compared with other common amorphous materials in Fig. 3c ^32–35^. Amorphous diamond shows the highest atomic density and the largest bulk modulus, clearly differentiating itself from all other amorphous materials.

## Discussion

Why can this quenchable amorphous diamond be synthesized? Treating glassy carbon at 18–21 GPa and 2100–2500 K using LVP was reported to yield crystalline diamond^18, 19^. In our experiments, using DAC coupled with laser heating, we went well beyond the P-T range of previous experiments and synthesized amorphous diamond from glassy carbon at 40–50 GPa and approximately 1800 K. We also observed nanocrystalline diamond formation when the temperature was set above ~2100 K. The starting glassy carbon material has a unique disordered structure with randomly orientated fragments of curved carbon sheets. The combination of relatively higher pressure and lower temperature used in our work provides energy for the *sp* ^2^-to-*sp* ^3^ transition, leading to the formation of a local tetrahedral structure; but these P-T conditions could not facilitate the long-range rearrangement of the tetrahedra required to form an ordered crystalline lattice from the disordered matrix of glassy carbon. Consequently, an *sp* ^3^-bonded amorphous structure (i.e. amorphous diamond) rather than nanocrystalline diamond was formed. As soon as the sample converted to transparent amorphous diamond during laser heating, its temperature dropped quickly due to the poor laser absorption of the transparent sample, inhibiting the high-temperature annealing-induced crystallization in the amorphous diamond. Carbon has a complex energy landscape with potential for finding local minima that can be metastably quenched. Therefore, amorphous diamond can be maintained at ambient conditions like crystalline diamond.

In summary, key factors leading to the formation of amorphous diamond are the starting materials (*sp* ^2^-bonded carbon with a disordered structure), the application of relatively high pressure (> 40 GPa) and a well-controlled moderate temperature (~1800 K). The combination of completely *sp* ^3^ bonding and a disordered atomic structure makes amorphous diamond an isotropic, dense, optically transparent, super strong, and potentially superhard material. More exceptional properties that combine the merits of crystalline diamond and amorphous materials are expected. Our results demonstrate that purely *sp* ^3^-bonded tetrahedral amorphous carbon can be synthesized and recovered to ambient conditions for fundamental studies and potential applications. In addition, although the P-T conditions in our work correspond to a region where crystalline diamond was believed to be thermodynamically stable and favorable, our results show that crystalline diamond is not necessarily the only possible product. It will be interesting to explore other forms of carbon over a greater P-T range using various carbon allotropes as precursors.

## Methods

### Synthesis of quenchable amorphous diamond

We cut type I glassy carbon balls (from Alfa Aesar) into approximately 40 micrometre diameter × 15 micrometre thick disks and loaded them into symmetric diamond anvil cells (DACs) with a culet size of 300 micrometres. The sample chamber was a 90–150 μm diameter hole drilled in a rhenium gasket. Sodium chloride was loaded as the pressure transmitting medium and thermal insulator. Pressure was measured by monitoring the shift in the R1 fluorescence line of a ruby ball, which was loaded along with the sample. A double-side YLF (wavelength 1064 nm) laser-heating system was used to heat the sample. Temperatures were determined by fitting the thermal radiation spectra of the heated sample to the Planck radiation function in a given wavelength range^36^. We first compressed the sample to 40–50 GPa, then heated it to approximately 1800 K. After laser-heating, the pressure was released, and the recovered sample was removed from the DAC for further characterization.

### XRD experiments

XRD experiments were performed in situ at high pressures and at ambient conditions on the recovered samples at beamlines 16 ID-B of the High Pressure Collaborative Access Team (HPCAT) and 13 ID-D of GeoSoilEnviroCARS (GSECARS), at the Advanced Photon Source (APS), Argonne National Laboratory (ANL). The wavelengths of the X-ray were 0.4066 Å and 0.2952 Å, respectively. A MAR165 charge-coupled device detector was used for data collection, and the software Dioptas was used to integrate the two-dimensional images^37^. The compressibility of the amorphous diamond was determined by high-pressure XRD experiments, during which a recovered amorphous diamond sample was compressed to ~25 GPa. Helium was loaded at GSECARS as pressure medium. Background scattering from the high-pressure environment was obtained by shining X-ray on the pressure medium beside the sample. The XRD signal from the sample was derived by subtracting the background scattering.

### HRTEM and EELS Experiments

HRTEM samples were prepared by crushing the recovered sample (tens of micrometre sized) and then dispersing these crushed powders onto a holey carbon grid. HRTEM experiments were carried out using the Argonne Chromatic-Aberration Corrected TEM (ACAT) with an image corrector to correct both spherical and chromatic aberration. An accelerating voltage of 80 kV was used to minimize the knock-out damage on the sample. EELS spectra around the carbon K-edge were collected to determine the chemical bonding in the samples. Low energy loss EELS (0–60 eV) was performed to determine the mass density of the sample. The energy resolution of the EELS measured from zero-loss peak was 0.8 eV.

### MD simulations

An *sp* ^3^-bonded tetrahedral amorphous carbon structure was obtained from a liquid state through extensive ab initio MD simulation with the density functional theory-based Vienna Ab-initio Simulation Package (VASP)^38^. The projector augmented wave potential (PAW) with a valence configuration of 2*s*2*p* and the generalized gradient approximation (GGA) were used in the simulation^39^. The kinetic energy cutoff was set to 400 eV, and the simulation was conducted on the gamma point only. Liquid carbon (288 atoms) was first equilibrated at 50 GPa and 7000 K for 20 ps in an NPH ensemble (constant number of atoms, pressure, and enthalpy) with each time step set to 2 fs. Equilibrated liquid carbon was then gradually cooled to 2000 K in 250 ps in the NPH ensemble by extracting heat from the system periodically, forming an amorphous solid (i.e. amorphous diamond) at 50 GPa. The as-obtained amorphous structure was further relaxed in an NPT ensemble at 300 K and 0 GPa for 20 ps. The structure factor of the amorphous diamond was computed based on the radial distribution function using the Baxter-Dixon-Hutchinson factorization method^40^. By setting a cutoff distance at 1.85 Å, the average CN of C was computed to be 3.95, which means approximately 95% of the carbon atoms were tetrahedrally coordinated. The structural differences between amorphous diamond and other amorphous carbon materials were discussed in Supplementary Note 1. We also conducted Wannier function analysis to examine the bonding nature in this structure^29, 30, 41^. The center of maximally localized Wannier functions (WFC) reflects the bonding of the atoms. Similar techniques have been used to characterize amorphous Si and C networks before^29, 42^. We have obtained the pair distribution of all the WFCs of occupied Wannier functions (Supplementary Fig. 5), in which almost all WFCs lie in the middle of two neighboring carbon atoms. By calculating the CN of WFC around C (0.5 Å < *r* < 1.0 Å), we derive the percentage of tetrahedral bonding to be 98%. The volume change of this amorphous structure under hydrostatic compression was also calculated, and its bulk modulus was derived by fitting the data to a third-order Birch-Murnaghan equation of state (Fig. 3b and Supplementary Fig. 6).

Based on the structural model, its EELS was treated with the all-electron ab initio code WIEN2K^43^, using the TELNES3 package^44^. The readers can refer to ref. ^44^ for the details of the theoretical approach. Consideration of the core-hole effect is found to be critical to obtain EELS comparable with the experimental results. The structural models were taken from our ab initio MD simulations as illustrated in the previous part. We obtained the EELS spectra of crystalline diamond, amorphous diamond, and glassy carbon through the following steps: (1) Self-consistent field (SCF) electronic calculations were performed on large supercells of crystalline diamond (216 atoms), amorphous diamond (288 atoms), and glassy carbon (288 atoms), respectively. We adopted the Perdew-Burke-Emzerhof type^45^ GGA for the exchange-correlation function, and the calculations were conducted in the non-magnetic state. The SCF calculations were performed on a 3 × 3 × 3 *k*-point mesh in the Brillouin zone. (2) The core-hole effect was considered by removing a certain amount of core electrons from an arbitrary atom in each system. To achieve the best match with the experiment, 0.5 electrons were removed from the 1 s state of an arbitrarily selected carbon atom, and treated as the background charge. With the new electronic configurations, SCF calculations were conducted to reach an energy convergence within 0.01 Ry. (3) After the SCF converged, EELS calculations were conducted by employing the TELNES3 method. The calculations were performed on a finer *k*-point mesh (5 × 5 × 5) with additional energy bands included (so that the calculated EELS have wider energy ranges).

Due to the highly intricate and demanding nature of the EELS calculations, the calculations were performed on 10 different atoms in glassy carbon or amorphous diamond to obtain average EELS spectra. For crystalline diamond the calculations were performed on only one atom due to atomic equivalency. The as-obtained EELS were plotted in Fig. 4c (*thin lines*), which were further broadened (*thick lines*) using a Gaussian smoothing window function (the window width is set as 1.2 eV). The calculated EELS of amorphous diamond featured by the absence of π bonding peak and broad post-edge band agrees well with the experimental results, further proving that the amorphous diamond synthesized in our study is *sp* ^3^ bonded.

### Data availability

The data that support the findings of this study are available from the corresponding authors upon request.

## Electronic supplementary material

Supplementary Information
