# Supplementary material for: Synthesis of quenchable amorphous diamond
Source: Nat Commun. 2017 Aug 22;8:322. doi: 10.1038/s41467-017-00395-w (PMC5567272; doi:10.1038/s41467-017-00395-w)
Supplement: Supplementary file 1 — Supplementary Information [file 41467_2017_395_MOESM1_ESM.pdf]

Title of file for HTML: Supplementary Information
Description: Supplementary Figures and Supplementary Note

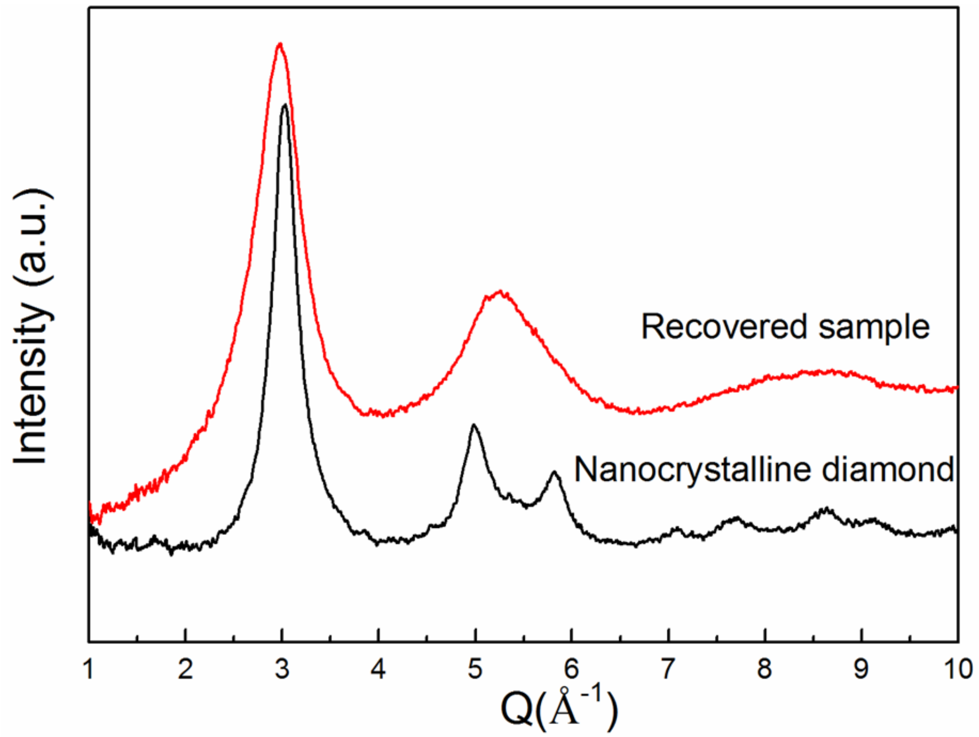

Supplementary Figure 1. **XRD patterns of the recovered sample and nanocrystalline diamond.** The first diffraction peak of the recovered sample has a position (2.964 Å^{-1}) close to that of diamond (111) peak (3.05 Å^{-1}), but with a much larger peak width (FWHM), i.e. 0.63 Å^{-1} for the recovered sample versus 0.32 Å^{-1} for the nanocrystalline (average grain size of ~2 nm) diamond (111) peak.

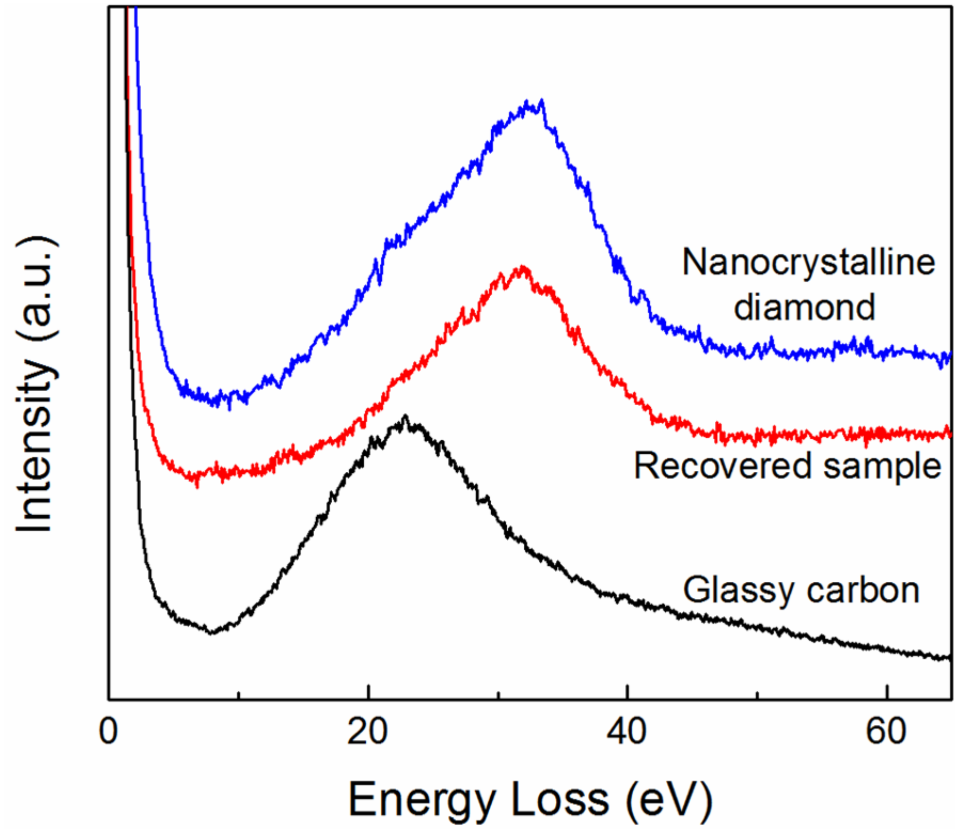

Supplementary Figure 2. **Low energy loss EELS.** The low energy loss EELS of the as-received glassy carbon (black), the recovered sample (red) and nanocrystallline diamond (blue).

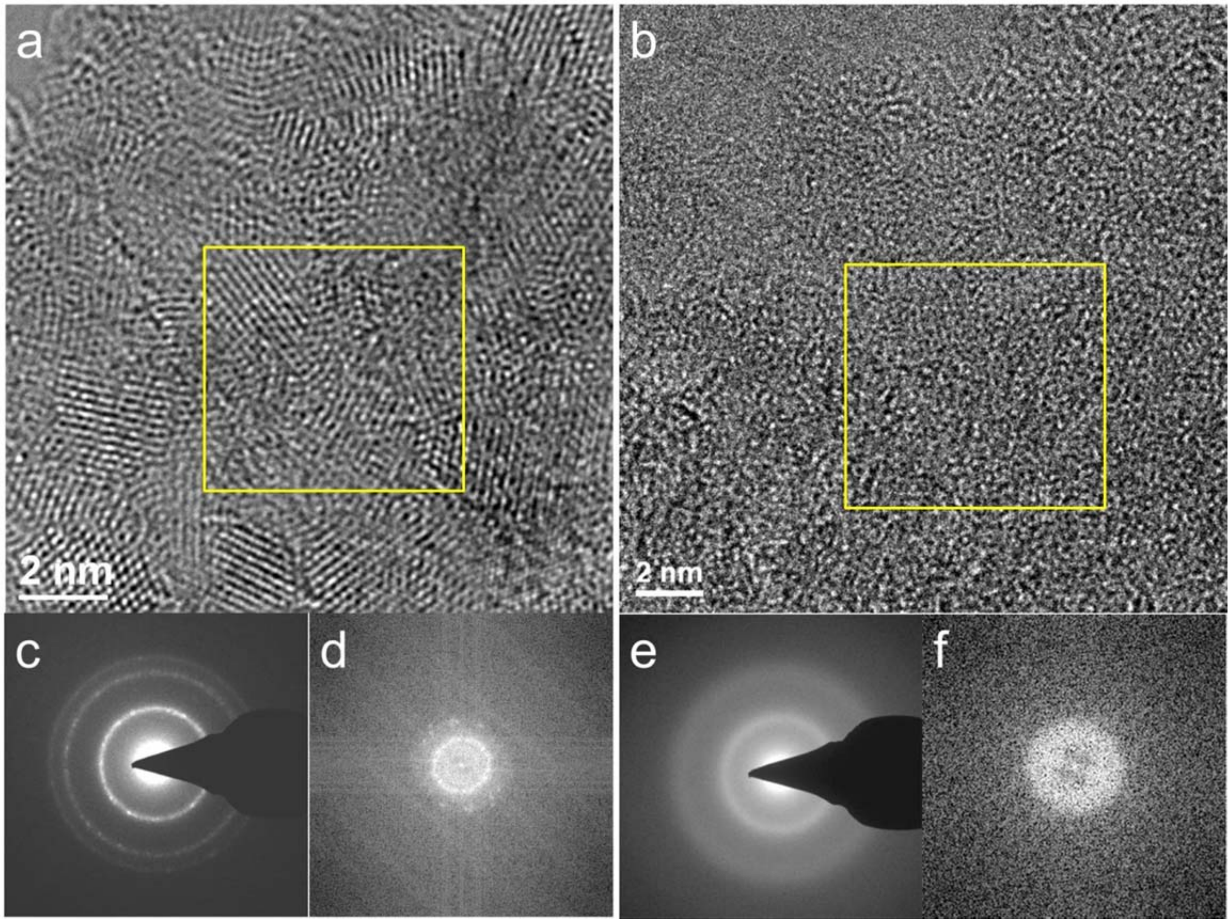

Supplementary Figure 3. **TEM studies of the recovered sample compared with nanocrystalline diamond** HRTEM image (a), SAED image (c), and selected area fast Fourier transformation (d) of nanocrystalline diamond with grain size 1-3 nm. The crystalline lattices are clearly visible, and the SAED shows sharp diffraction rings. HRTEM image (b), SAED image (e), and selected area fast Fourier transformation (f) of the recovered sample confirm its amorphous structure. The areas for Fourier transformation are marked by yellow squares.

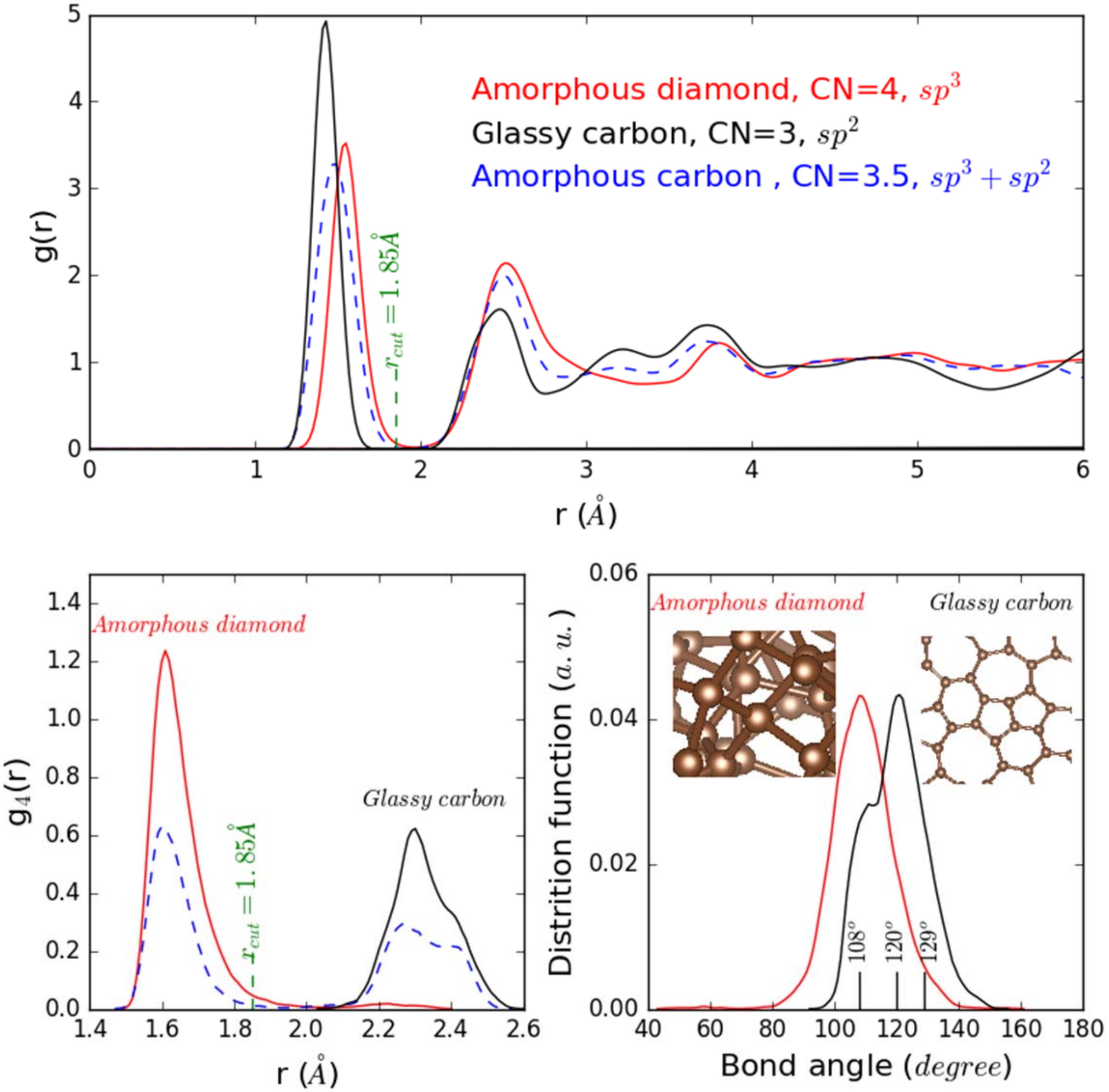

Supplementary Figure 4. **Structural differences between amorphous diamond, glassy carbon and amorphous carbon derived from first-principles MD simulations. a**, Radial distribution functions (RDF) of the amorphous diamond (red), glassy carbon (black), and amorphous carbon (blue dashed line). The characteristic C-C bond-length is 1.43 Å for glassy carbon versus 1.55 Å for the amorphous diamond. **b**, The RDF of the fourth nearest neighbors of C, $g_4(r)$. **c,** Bond-angle distribution of the carbon atoms within the first atomic shell for amorphous diamond (red) and glassy carbon (black). For the amorphous diamond, the bond-angle distribution is centered at 109.5°, implying its tetrahedral structure. The angles marked in the figure correspond to 5-, 6-, 7-member rings of the glassy carbon (see the inset for a sketch of glassy carbon).

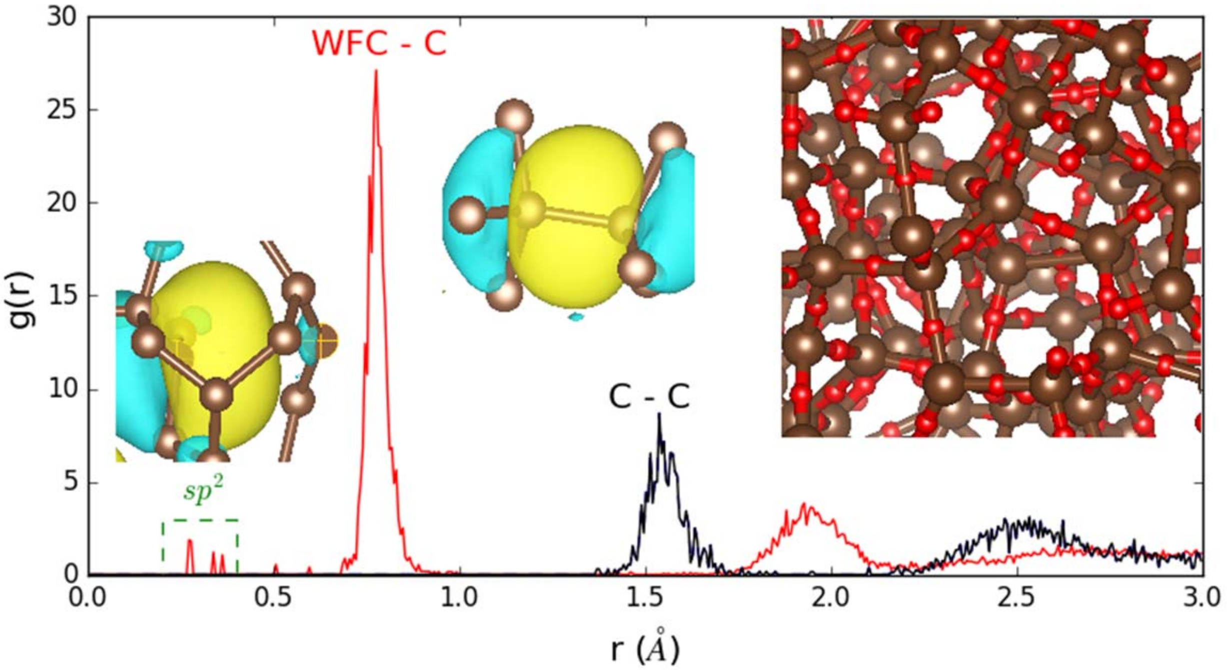

Supplementary Figure 5 Pair distribution functions of Wannier function centers (WFC) and carbon atoms in the computed amorphous structure. The rightmost inset shows the configuration of the computed WFCs and carbon network, from which it can be seen that most WFCs (red balls) lie between two neighboring carbon atoms. The inset in the middle shows an isosurface of a maximally localized Wannier function, manifesting the chemical bonding of two C atoms (the WFC is not shown). The inset on the left shows the WFC is close to one of the carbon atoms (< 0.5 Å), and is not situated in-between any two neighboring C atoms, indicating the “delocalized” Wannier function (with a large spread) is associated with sp^2 bonding. The spikes on the WFC-C pair distribution function in the short range (r < 0.5 Å) can be ascribed to non-tetrahedral bonding of C atoms, which can be considered as defects in the tetrahedral network. The fraction of sp^3 bonding is estimated to be 98%, based on the number of WFC around each C atom.

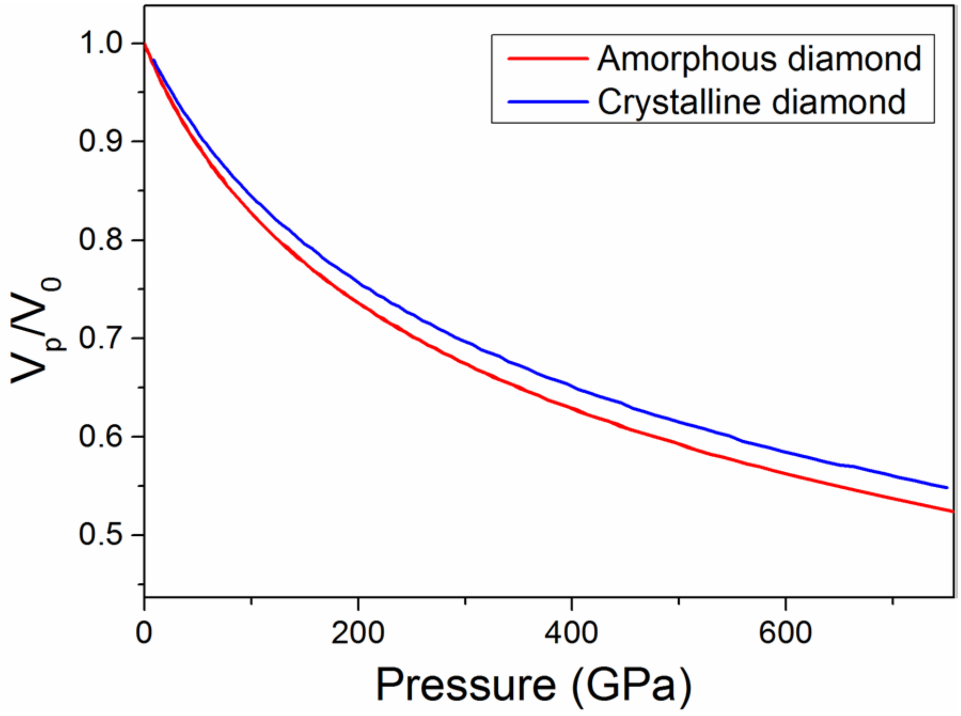

Supplementary Figure 6. **Equation of state.** Equation of state of amorphous diamond and crystalline diamond derived from first-principles MD simulations.

SUPPLEMENTARY NOTE 1

Structural differences between amorphous diamond and other amorphous carbon materials from first-principles MD simulations

Our *ab initio* MD simulation reveals that tetrahedral amorphous carbon (i.e. amorphous diamond) can be synthesized at high pressure (e.g. 50 GPa). Using low pressures in simulation (e.g. below 10 GPa), instead of amorphous diamond, we obtained glassy carbon (cooling rate ~$2x10^{13}$ K s^{-1}) or an amorphous carbon structure with ~ 50% tetrahedral bonding (cooling rate ~$5x10^{14}$ K s^{-1}) from liquid carbon. These results confirm that a relatively high pressure is one of the key factors to form amorphous diamond.

For structural analysis, the radial distribution functions (RDF), *g(r)*, of the three as-obtained amorphous structures of carbon are plotted in Supplementary Figure 4. Setting a cutoff distance at 1.85Å, we obtained the coordination number (CN) for the amorphous diamond as 3.95, in contrast to CN=3.0 for the glassy carbon and CN=3.5 for the amorphous carbon. The CN=3.0 of the glassy carbon is consistent with its sp^2 bonding.

The structural differences between amorphous diamond and glassy carbon can be clearly revealed by plotting the RDF of the fourth nearest neighbors $g_4(r)$ of the carbon atoms, where $\int_0^\infty r g_4(r) \mathrm{d}r = 1$. In the amorphous diamond, most of the fourth nearest neighbors belong to the first atomic shell (suggesting tetrahedral bonding), whereas in glassy carbon, the fourth nearest neighbors belong to the second shell. In terms of bond-angle distributions, the amorphous diamond has a bond-angle distribution function centered at 109.5^{o}, which is characteristic of tetrahedral bonding. These results suggest that our structural model of amorphous diamond is a nearly perfect tetrahedral amorphous structure.
